# The Association between Exposure to Environmental Bisphenol A and Gonadotropic Hormone Levels among Men

**DOI:** 10.1371/journal.pone.0169217

**Published:** 2017-01-13

**Authors:** Hong Liang, Wenping Xu, Jianping Chen, Huijuan Shi, Jiang Zhu, Xiaoqin Liu, Jian Wang, Maohua Miao, Wei Yuan

**Affiliations:** 1 Department of Reproductive Epidemiology and Social Science, Key Lab. of Reproduction Regulation of NPFPC, SIPPR, IRD, Fudan University, Shanghai, China; 2 Shanghai Key Lab of Chemical Biology, School of Pharmacy, East China University of Science & Technology, Shanghai, China; 3 Department of Reproductive Biology, Key Lab. of Reproduction Regulation of NPFPC, SIPPR, IRD, Fudan University, Shanghai, China; 4 Department of Orthogenics and Genetics, Population and Family Planning Institute of Guizhou Province, Guiyang, China; 5 Department of Public Health, Aarhus University, Aarhus, Denmark; Nanjing Medical University, CHINA

## Abstract

Bisphenol A (BPA) is an extensively used chemical with endocrine disrupting properties. Although animal and in vivo studies have suggested possible effects of BPA on levels of gonadotropic hormones, human studies are limited and inconclusive. The study examined whether environmental BPA exposure was associated with gonadotropic hormones levels in men. A total of 560 men aged 18–55 years were recruited from Sandu County, Guizhou Province, China. We collected urine samples for measurement of BPA, and blood samples for measurement of reproductive hormones. We examined serum levels of follicle-stimulating hormone (FSH), luteinizing hormone (LH), and total testosterone (T). Relative risk (RR) was obtained by log-binominal regression to explore the association between urinary BPA level and hormone levels. BPA was detected in 70.4% of urine samples, with a geometric mean of 0.50 μg/gCr. Men with detectable levels of BPA had a 1.52-fold increased risk of having a high LH level (>75^th^ percentile) when compared with men with undetectable levels of BPA, after adjustment for potential confounders (95% confidence interval (CI): 1.04–2.21). The association persisted and slightly intensified among current smokers (adjusted RR (aRR) = 1.76, 95%CI: 1.05–2.95), while it weakened among non-smokers (aRR = 1.17, 95%CI: 0.69–1.96). Urinary BPA level was associated with an increased FSH level among smokers (aRR = 1.64, 95%CI: 1.01–2.67). Urinary BPA level was inversely associated with total T level among males with body max index (BMI) ≥25 kg/m^2^ although this association was of borderline significance (aRR = 0.52, 95%CI: 0.26–1.05). In conclusion, environmental exposure to BPA was associated with increased serum levels of LH and FSH in male smokers, along with decreased serum levels of total T in men with BMI≥25 kg/m^2^. These findings suggest that the effects of environmental BPA exposure on hormone levels might be modified by smoking and BMI.

## Introduction

Bisphenol A (BPA), an exogenous endocrine-disrupting chemical, is extensively used in many consumer products and can be found in the resin linings of cans used for food and beverages, plastics containers, baby bottles, dental sealants, and thermal receipts. BPA-containing product can leach the chemical into food, water, and ecosystems[1], leading to widespread human exposure. In fact, BPA was detected in more than 90% of urine samples among the general population of the USA[2] and other countries[3–5].

Accumulating evidence suggests that BPA binds with estrogen receptor or androgen receptor and has both estrogenic and anti-androgenic effects[6, 7]. Animal studies have reported the adverse effects of BPA on male reproductive health, including sperm production and quality, steroidogenesis, male urinary tract development, and sexual dysfunction[8]. Regarding steroidogenesis, rodent studies indicate that BPA exposure decreases testosterone (T) levels in the serum or testis, and also affects serum levels of luteinizing hormone (LH) and follicle-stimulating hormone (FSH)[9–12].

Evidence supporting an association between environmental BPA exposure and gonadotropic hormone levels in human is limited and inconclusive[4, 13–17]. The reason for the inconclusive findings may be due to differences in BPA exposure level, with geometric means (GM) of urinary BPA varying from 1.30 to 3.59 μg/L in men without occupational exposure. Additionally, characteristic differences between members of the study population, such as men with occupational exposure, fertile men, or infertile men, may explain these inconsistencies.

Two previous studies have reported that the GM of urinary BPA concentration in the general population in China is 0.87 μg/L[18] and 1.01 μg/L[19]. In the present study, the study population was derived from Sandu County, Guizhou Province, which is located in a mountainous area of West China and is much less industrialized. The GM of urinary BPA concentration in this population was 0.44μg/L, which is slightly lower than that reported by previous studies in China[18, 19] and much lower than those in developed countries (1.30–3.59 μg/L)[4, 14, 16, 17]. Compared with more industrialized areas, the minimal pollution caused by plastic products and the relatively simple lifestyle of the residents may explain the low BPA exposure level in the local population.

Accumulating evidence shows that threshold values of BPA concentration on human effects are much lower than the recommended safety limit. BPA may have effects on human reproduction even at a lower concentration. In this study we examined whether exposure to environmental BPA at a lower level has adverse effects on serum concentrations of FSH, LH, and total T among males in Sandu County, Guizhou Province.

## Materials and Methods

### Study population

We conducted a cross-sectional study based on a primary health program that aimed to promote the reproductive health of couples in less developed areas by providing free medical counseling as well as free semen quality assays for couples of childbearing age. This study was conducted from July to August, 2012 in Sandu County (Autonomous County of Shui nationality), Guizhou Province, China. Males aged 18–55 years who had at least one child were eligible for the present study and were invited to participate.

A total of 774 eligible men agreed to participate (participation rate of approximately 75%). Among them, 588 men provided blood samples for the hormone assay and urine samples for the BPA assay. We excluded 28 men who did not provide either in-person information or information at the physical examination. A total of 560 men (72.4% of eligible males) were ultimately included in the present analysis.

### In-person information and bio-sample collections

We obtained information on demographics, lifestyle factors (smoking, alcohol and caffeine consumption), history of exposure to chemicals, and reproductive history via an in-person interview. Individuals who reported smoking at least one cigarette per day for the last six months were considered as current smokers. Individuals who reported drinking an alcoholic beverage (beer, wine, and liquor) at least once a week for the last six months were considered as current drinkers. History of exposure to chemicals was defined as contacting chemical substances at least half an hour each day for at least seven days each year. Body height and weight were measured with the subjects barefoot and clad only in light underwear, following the recommendations of the Committee on Nutritional Anthropometry of National Health and Nutrition Examination Survey[20]. Body mass index (BMI) was calculated as weight (kg) divided by height squared (m^2^).

We collected a single-spot urine sample from each subject in a polypropylene cup when they visited the clinic. Venous blood samples were also drawn at the same time, and the serum was separated and frozen at -20°C. Both urine and serum samples were shipped to the laboratory at the Shanghai Institute of Planned Parenthood Research (Shanghai, China) on dry ice and stored at -80°C for further analysis.

### Urinary BPA measurements

The urine samples were analyzed at the collaborative laboratory of East China University of Science and Technology (Shanghai, China). We measured urinary concentrations of total BPA (free plus conjugated species) using modified high-performance liquid chromatography (HPLC) as described by He et al[21]. Briefly, urine samples were treated with phosphorous acid buffer/ β-glucuronidase for hydrolyzation, and were subsequently extracted twice using ether (HPLC grade, Dikma). The supernatants were collected and evaporated with nitrogen gas. The residue was dissolved in 60% acetonitrile and analyzed using HPLC equipment. The limit of detection (LOD) of BPA in the present study was 0.12μg/L.BPA levels below the LOD were assigned a value of LOD divided by the square root of 2, based on a conventionally accepted practice.[22] Adjustment for creatinine was performed to account for urine volume.

### Serum hormones analysis

We determined serum FSH, LH and total T with an electrochemiluminescence immunoassay, using kits provided by Roche Diagnostics GmbH (Mannheim, Germany) (FSH: intraassay coefficients of variation [CV] <4.5%, interassay CV<5.3%, sensitivity <0.100 IU/L; LH: intraassayCV <2.2%, interassay CV<5.2%, sensitivity <0.100 IU/L; T: intraassay CV <4.7%, interassay CV<8.4%, sensitivity <0.025 ng/mL).

### Statistical analysis

Descriptive statistics of urinary BPA concentration and serum hormone levels were tabulated. We calculated GMs and percentiles of original and creatinine-adjusted BPA concentration. The natural log of serum hormone levels was used to achieve normal distributions. Then, an ANOVA test was used to test for differences in hormone levels according to BPA categories.

We used relative risk (RR) and 95% confidence interval (CI) in a log binominal regression model to examine the associations between BPA level (detected or not) and higher hormone levels (>75^th^ percentile). As the following variables might be associated with BPA level and hormone levels, they were adjusted for accordingly: age (<25, 25–29, 30–34, 35–39 and ≥40 years), alcohol consumption (current drinker vs. not current drinker), BMI (<18.5, 18.5–24.9, and ≥25 kg/m^2^), history of exposure to chemicals (Yes, No), and ethnicity (Shui, Bouyei, Miao, and other nationalities). We further divided the men for whom urinary BPA was detected, into three categories according to tertiles of creatinine-adjusted BPA level to examine the dose-response relationships between increasing urinary BPA concentration and hormone levels.

To examine a potential modifying effect on the association between urine BPA and hormone levels, we performed stratified analyses according to two strong predictors of hormone levels, smoking status and BMI categories[23–25]. Considering that effects of BPA on hormones may be associated with men’s history of exposure to chemicals, we performed sensitive analysis by excluding those with history of exposure to chemicals (n = 29) and those without the information (n = 15). All data handling and statistical analyses were performed in SAS (version 9.2) (SAS Institute, Inc., Cary, North Carolina).

### Ethics

The study was reviewed and approved by the ethics committee board of Shanghai Institute of Planned Parenthood Research (IRB00008297). All participants gave written informed consent before participating in the study.

## Results

The average age of the participants was 32.2±6.5 years (range: 19–54 years). Table 1 presents descriptive data on male reproductive hormones and urinary BPA concentrations before and after creatinine adjustment. BPA was detected in 70.4% of urine samples, with a GM of 0.50 μg/gCr. Among participants with detectable BPA levels, the GMs of original and creatinine-adjusted BPA levels were 0.87 μg/l and 0.96 μg/gCr, respectively. Table 2 shows urinary creatinine-adjusted BPA levels and hormone levels according to the characteristics of participants. Men with a higher BMI had higher BPA levels. Those of Bouyei nationality had higher BPA levels than those of other nationalities. Higher levels of FSH, LH, and T were found in men aged at ≥40 years. Men of Miao nationality had a lower level of FSH and T compared with Bouyei nationality. Those with a BMI ≥25 kg/m^2^had a lower T level.

**Table 1:** Concentrations of urinary bisphenol A (BPA) and serum gonadotropic hormones (n = 560).

	Geometric mean (95%CI)	Percentile				
		5th	25th	50th	75th	95th
BPA (μg/L)	0.44 (0.39–0.50)	0.08	0.08	0.38	1.26	6.96
Creatinine-adjusted BPA^a^(μg/gCr)	0.50(0.44–0.58)	0.06	0.14	0.41	1.36	8.74
**Hormone**^b^						
FSH(IU/L)	4.67(4.49–4.87)	2.21	3.50	4.80	6.30	9.85
LH(IU/L)	4.82(4.64–5.00)	2.20	3.73	5.01	6.60	9.30
T(ng/mL)	5.18(5.02–5.34)	2.68	4.14	5.35	6.82	9.36

^a^ 10 missing value;

^b^ FSH: Follicle-stimulating hormone, LH: Luteinizing hormone, T: Total testosterone. The mean (standard deviation) of FSH, LH, and T was 5.21(2.53), 5.30(2.25), and 5.54(2.01) IU/L, respectively.

**Table 2:** Characteristics of the study population and urinary bisphenol A (BPA) concentrations.

Characteristics	N(%)	Geometric mean(95%CI)			
		Creatinine-adjusted BPA^a^ (μg/gCr)	FSH(IU/L)	LH(IU/L)	T(IU/L)
Age group (years)					
<25	67(12.0)	0.57(0.39–0.84)	4.37(3.90–4.89)	4.87(4.42–5.37)	4.92(4.56–5.31)
25–29	147(26.3)	0.46(0.36–0.61)	4.35(4.07–4.65)	4.60(4.28–4.94)	4.93(4.61–5.28)
30–34	149(26.6)	0.54(0.42–0.70)	4.68(4.35–5.02)	4.82(4.45–5.24)	5.13(4.81–5.48)
35–39	119(21.2)	0.42(0.32–0.56)	4.81(4.37–5.29)	4.74(4.37–5.14)	5.33(4.98–5.70)
≥40	78(13.9)	0.61(0.40–0.94)	5.45(4.79–6.20)	5.33(4.81–5.90)	5.78(5.37–6.23)
Education^b^					
≤Primary school	215(38.9)	0.53(0.47–0.66)	4.77(4.46–5.09)	4.89(4.61–5.19)	5.06(4.80–5.34)
Junior high school	266(48.1)	0.51(0.41–0.62)	4.65(4.40–4.91)	4.67(4.41–4.94)	5.36(5.14–5.60)
≥Senior high school	72(13.0)	0.44(0.31–0.64)	4.60(4.04–5.24)	5.34(4.82–5.91)	4.88(4.43–5.37)
Race/ethnicity					
Shui nationality	284(50.7)	0.43(0.36–0.51)	4.60(4.33–4.88)	4.91(4.64–5.19)	5.26(5.02–5.51)
Bouyei nationality	152(27.1)	0.75(0.56–1.01)	5.01(4.67–5.38)	4.82(4.51–5.15)	5.36(5.06–5.67)
Miao nationality	96(17.1)	0.43(0.33–0.56)	4.37(4.01–4.76)	4.56(4.19–5.03)	4.78(4.41–5.19)
Other nationalities	28(5.0)	0.54(0.29–1.01)	4.80(4.03–5.72)	4.71(3.83–5.78)	4.82(4.29–5.41)
Current smoker^b^					
No	238(43.2)	0.45(0.37–0.55)	4.53(4.25–4.82)	4.84(4.57–5.11)	5.02(4.77–5.29)
Yes	313(56.8)	0.56(0.46–0.67)	4.82(4.58–5.08)	4.85(4.60–5.11)	5.32(5.11–5.53)
Alcohol intake^b^					
No	215(39.7)	0.53(0.42–0.66)	4.85(4.56–5.15)	4.96(4.67–5.27)	5.36(5.07–5.67)
Yes	327(60.3)	0.49(0.41–0.58)	4.54(4.30–4.79)	4.76(4.52–5.00)	5.09(4.90–5.29)
Body mass index (kg/m^2^)					
<18.5	26(4.6)	0.32(0.17–0.61)	4.38(3.58–5.34)	4.68(3.69–5.94)	5.38(4.69–6.16)
18.5–24.9	369(65.9)	0.49(0.42–0.57)	4.79(4.56–5.02)	4.78(4.55–5.01)	5.46(5.26–5.67)
≥25	165(29.5)	0.59(0.44–0.77)	4.48(4.14–4.83)	4.93(4.63–5.24)	4.57(4.31–4.85)
History of exposure to chemicals^b^					
No	516(94.7)	0.50(0.44–0.58)	4.70(4.50–4.90)	4.82(4.63–5.01)	5.19(5.02–5.37)
Yes	29(5.3)	0.65(0.34–1.26)	4.71(4.09–5.43)	5.20(4.37–6.18)	5.00(4.30–5.82)

^a^ 10 missing values;

^b^ There are 7, 9, 18, and 15 missing values in education, current smoker, alcohol intake, and history of exposure to chemicals respectively.

Table 3 presents the average concentrations of reproductive hormones by BPA category. We divided the subjects into four groups according to BPA level (Undetectable BPA; and lowest, middle, and highest tertiles of detectable BPA). Participants with detectable BPA had slightly higher concentrations of FSH and LH; however, none of the differences in hormone levels among the four groups were statistically significant.

**Table 3:** Concentrations of serum gonadotropic hormones (geometric mean (95%CI)) according to creatinine-adjusted BPA category.

Hormones^a^	BPA undetected(n = 166)	BPA detected		
		Lowest tertile(n = 130)	Middle tertile(n = 126)	Highest tertile(n = 128)
FSH(IU/L)^b^	4.53(4.25–4.83)	4.68(4.32–5.07)	4.73(4.34–5.16)	4.80(4.35,5.29)
LH(IU/L) ^b^	4.60(4.31–4.90)	5.08(4.69–5.51)	5.05(4.68–5.45)	4.69(4.29–5.13)
T(ng/mL) ^b^	5.12(4.83–5.43)	5.22(4.87–5.60)	5.16(4.83–5.53)	5.26(4.93–5.60)

^a^ FSH: Follicle-stimulating hormone, LH: Luteinizing hormone, T: Total testosterone;

^b^ There are no statistically differences of hormones concentrations among four groups of BPA levels. ANOVA test for FSH: F = 0.38, P = 0.7694; for LH: F = 1.76,P = 0.1528; for T: F = 0.14,P = 0.9379.

To examine the association between BPA exposure and higher hormone levels (>75^th^ percentile), we estimated RRs using log binominal regression. Compared with men with undetectable urinary BPA, those with detectable BPA had a 1.47-fold higher risk of an elevated LH level. After adjusting for potential confounders, the association remained similar (adjusted RR (aRR) = 1.52, 95%CI: 1.04–2.21). Neither FSH nor total T levels showed a statistically significant association with urinary BPA level. We did not observe any dose-response relationships between increasing BPA level and hormone levels (Table 4). When the analyses were performed among those without history of exposure to chemicals, the results only changed slightly (S1 Table).

**Table 4:** Associations between urinary bisphenol A (BPA) and serum hormones^a^.

	BPA detection	N	%(Hormone>P_75_)	Crude RR(95%CI)	Adjusted RR ^b^(95%CI)
FSH(IU/L)	No	166	22.3	Ref	Ref
	Yes	394	26.1	1.17(0.84–1.63)	1.23(0.89–1.72)
	Lowest tertile^C^	130	24.6	1.08(0.72–1.63)	1.18(0.79–1.77)
	Middle tertile	126	25.4	1.12(0.75–1.67)	1.23(0.82–1.85)
	Highest tertile	128	27.3	1.24(0.84–1.83)	1.19(0.80–1.75)
LH(IU/L)	No	166	17.5	Ref	Ref
	Yes	394	25.6	**1.47(1.01–2.13)**	**1.52(1.04–2.21)**
	Lowest tertile	130	24.6	1.40(0.90–2.17)	1.48(0.95–2.30)
	Middle tertile	126	25.4	1.44(0.93–2.23)	1.47(0.84–2.29)
	Highest tertile	128	27.3	1.55(1.01–2.38)	1.49(0.96–2.31)
T(ng/mL)	No	166	28.3	Ref	Ref
	Yes	394	24.9	0.88(0.65–1.18)	0.83(0.62–1.12)
	Lowest tertile	130	30.0	1.08(0.76–1.54)	1.00(0.70–1.43)
	Middle tertile	126	23.8	0.86(0.58–1.27)	0.90(0.62–1.32)
	Highest tertile	128	21.1	0.76(0.50–1.14)	0.70(0.47–1.06)

^a^ FSH: Follicle-stimulating hormone, LH:Luteinizing hormone, T: Total testosterone

^b^RR: relative risk; adjusted for age, BMI, nationality, alcohol intake, and history of chemical exposure.

^C^ There are 10 missing values in creatinine-adjusted BPA concentration due to 10 missing values in creatinine measurements. Therefore, the sum of 3 tertiles was 10 less than the number of BPA exposed men.

Among current smokers (the arithmetic mean (SD) of smoking duration = 12.8 (6.9) years), the association between urinary BPA and serum LH persisted and intensified (aRR = 1.76, 95%CI: 1.05–2.95), while it decreased and became non-significant among non-smokers (aRR = 1.17, 95%CI: 0.69–1.96). Urinary BPA was associated with an increased risk of a high FSH level among smokers (aRR = 1.64, 95%CI: 1.01–2.67) but not in non-smokers (aRR = 0.84, 95%CI: 0.52–1.37). There was no statistically significant association between urinary BPA and total T levels regardless of the smoking status (Table 5).

**Table 5:** Associations between urinary bisphenol A (BPA) and serum hormones ^a^, stratified by smoking status and body mass index (BMI).

	BPA detection	N	%(Hormone>P_75_)	Crude RR(95%CI)	Adjusted RR ^b^(95%CI)
Current smokers					
FSH(IU/L)	No	87	19.5	Ref	Ref
	Yes	226	30.5	**1.56(0.97–2.50)**	**1.64(1.01–2.67)**
LH(IU/L)	No	87	16.1	Ref	Ref
	Yes	226	27.0	**1.68(0.99–2.84)**	**1.76(1.05–2.95)**
T(ng/mL)	No	87	32.2	Ref	Ref
	Yes	226	25.2	0.78(0.54–1.14)	0.79(0.55–1.14)
Non-smokers					
FSH(IU/L)	No	75	26.7	Ref	Ref
	Yes	163	20.9	0.78(0.48–1.26)	0.84(0.52–1.37)
LH(IU/L)	No	75	20.0	Ref	Ref
	Yes	163	23.9	1.20(0.70–2.03)	1.17(0.69–1.96)
T(ng/mL)	No	75	24.0	Ref	Ref
	Yes	163	24.5	1.02(0.63–1.66)	0.95(0.57–1.56)
BMI<25(kg/m^2^)					
FSH(IU/L)	No	112	24.1	Ref	Ref
	Yes	283	27.9	1.16(0.79–1.69)	1.24(0.84–1.84)
LH(IU/L)	No	112	17.0	Ref	Ref
	Yes	283	25.8	**1.52(0.96–2.40)**	**1.60(1.01–2.54)**
T(ng/mL)	No	112	30.4	Ref	Ref
	Yes	283	30.0	0.99(0.71–1.38)	0.96(0.69–1.34)
BMI≥25(kg/m^2^)					
FSH(IU/L)	No	54	18.5	Ref	Ref
	Yes	111	21.6	1.17(0.60–2.26)	1.11(0.59–2.09)
LH(IU/L)	No	54	18.5	Ref	Ref
	Yes	111	25.2	1.36(0.71–2.60)	1.12(0.58–2.17)
T(ng/mL)	No	54	24.1	Ref	Ref
	Yes	111	11.7	**0.49(0.24–0.98)**	**0.52(0.26–1.05)**

^a^ FSH: Follicle-stimulating hormone, LH: Luteinizing hormone, T: Total testosterone

^b^RR: relative risk; adjusted for age, BMI, nationality, alcohol intake, and history of chemical exposure.

After stratification by BMI, we observed that the association of BPA levels with LH levels persisted among those with BMI<25 kg/m^2^ (aRR = 1.60, 95%CI: 1.01–2.54) and weakened among men with BMI≥25 kg/m^2^ (aRR = 1.12, 95%CI: 0.58–2.17). Urinary BPA levels were negatively associated with total T levels among men with BMI≥25 kg/m^2^ with borderline significance (aRR = 0.52, 95%CI: 0.26–1.05), and this association was not observed among those with BMI<25 kg/m^2^ (aRR = 0.96, 95%CI: 0.69–1.34). No statistically significant association between urinary BPA levels and FSH levels was observed for BMI categories (Table 5).

## Discussion

This study was the first report to explore relationships between exposure to environmental BPA and serum gonadotropic hormone levels in a relatively large sample of Chinese men. In the present study, exposure to low environmental levels of BPA (GM: 0.50 μg/gCr) was associated with a higher serum LH, especially among current smokers and men with BMI<25 kg/m^2^. Moreover, BPA exposure was associated with a higher serum FSH level among current smokers and a lower serum total T level among men with BMI≥25 kg/m^2^.

In a previous study, Cha et al.[13] observed that occupational exposure to BPA was associated with increased LH levels, but not with FSH levels. Moreover, Lassen et al.[4] reported that BPA concentrations above the lowest quartile were associated with higher LH levels compared with the lowest quartile, but not with FSH levels among 308 young men from the general population. A study conducted by Mendiola et al.[17] among 375 partners of pregnant women also reported a suggestive positive association with LH levels, but not FSH level. In addition, Liu et al.[15] observed no association between BPA exposure and FSH levels among 427 male workers. However, Meeker et al.[16] found a positive association with FSH levels but not LH levels among 167 men from an infertility clinic. Likewise, Hanaoka et al.[26]found a mild correlation of BPA exposure with FSH but not LH levels among 84 occupational male workers. Regarding the association with T level, most of the above-mentioned studies[13, 15–17, 26] demonstrated that BPA exposure was not associated with the T level, except for the studies of Lassen et al.[4] and Galloway et al.[14] which reported a significant positive association.

There are some discrepancies between our results and those mentioned above. The inconsistent findings may partly be due to differences in the characteristics and sizes of the study populations. For instance, the participants in the present study were men who had at least one child, and current smokers accounted for 58.6% of the current participants which was much higher than in previous studies[14, 15, 17]. Moreover, BPA exposure levels are different among studies. The GM of BPA levels (0.44μg/L) was much lower than in previous studies (1.30–3.59μg/L). In the present study, BPA was detected only in 70% of the participants with a LOD of BPA 0.12 μg/L. We examined the effect of BPA exposure by comparing detected with undetected BPA, while previous studies, where BPA was detectable in more than 90% of the participants with a LOD of 0.12 to 0.50 μg/L, applied linear regression or used the lowest quartile of BPA levels as reference. These disparate approaches may lead to divergent findings.

The stronger associations of BPA levels with LH and FSH levels among smokers in the current study suggest there may be a synergetic effect of BPA and tobacco on serum hormone levels. Current evidence indicates that smoking is associated with increases in plasma LH and T levels in humans,[25, 27] and suggests that heavy smoking further increases the FSH level[27]. One possible explanation is that tobacco may act on the hypothalamus to increase gonadotropin-releasing hormone release or may directly act on the hypophysis, thus leading to increased hormone levels[25]. By acting as an anti-estrogen, BPA might block the estrogenic effect by competing with endogenous estrogen[28, 29]. BPA may competitively inhibit the binding of natural estrogen to estrogen receptors at the hypothalamic or pituitary level. This would attenuate the negative feedback of circulating estrogen on LH and FSH release, resulting in higher circulating concentrations of LH and FSH. In addition, BPA may affect the functions of Sertoli cell in the seminiferous tubules in animal study[11], where inhibin B is synthesized and secreted[30], thus decrease the levels of inhibin B. Epidemiological studies also suggested urinary BPA may be inversely associated with serum inhibin B levels[4, 16]. Since FSH secretion is regulated at the pituitary level by inhibin B, the increased FSH levels may also be explained by the negative feedback of decreased serum inhibin B. However, further studies are needed to clarify the mechanisms by which smoking and BPA work together to produce the synergetic effect. In the current study, a concurrent increase in T levels was not shown with increased LH and FSH levels. The reason needed to be clarified. Based on an epidemiology study, BPA may influence the levels of SHBG and free androgen index rather than total T levels[17]. We speculate that the signal of increased FSH and LH may be insufficient to trigger the change of serum total T level.

In the present study, environmental BPA exposure was associated with a decreased serum total T concentration only among men with BMI≥25 kg/m^2^. Current evidence shows that BMI is inversely related to the serum total T level[23, 31, 32]. Low serum levels of total T among people with excess body weight may be attributed to an increase in the activity of adipocellular aromatase, an enzyme that converts androgens into estrogens[33], direct action of adipocyte-derived hormones such as leptin on Leydig cells[34, 35], and decreased sex hormone binding globulin (SHBG) binding capacity[36]. In vitro and rodent studies have shown that BPA may inhibit testicular functions in pubertal and adult rodents through changes in the expression of steroidogenic enzymes[9, 11, 37], which then affect steroid synthesis (T production) and circulating steroid levels. The findings suggest that the inhibitory effect of BPA on T may be enhanced by excess body weight. However, the observed inverse association between BPA exposure and T level was not paralleled by a significant increase in serum LH levels. Higher levels of circulating estradiol due to excess body weight may suppress a compensatory rise in LH, since the effect of androgens on LH secretion depend on a balance of signaling through both the estrogen and androgen receptors[38].

There is less pollution in Sandu county, Guizhou Province because it is less industrialized. These findings were, therefore, less likely to be confounded by unmeasured co-exposures, especially other environmental endocrine disruptors. The relatively large sample size in the study provided an opportunity to adjust for some potential confounders and allowed us to perform stratified analyses to examine how BMI and smoking modify the association between BPA exposure and hormone levels.

Our study should also be interpreted in light of the limitations. First, a single-spot urine sample was used to categorize BPA exposure level. BPA is rapidly metabolized within several hours in humans, therefore, the single urine sample may only reflect the most recent BPA exposure. However, a study by Mahalingaiah et al.[39] showed that a single-spot urine sample could predict a subject’s tertile categorization with moderate sensitivity. Moreover, the misclassification due to a single-spot sample would be non-differential, and thus may dilute any actual association. Second, when stratifying the analyses among smokers and non-smokers, we depended on the self-reported smoking status to classify the participants without measuring their serum cotinine levels. Thus, we could not refine the analyses according to the cotinine levels. Third, men who participated in the study may be more concerned about their reproductive health or more likely to have current reproductive problems, which may cause selection bias. However, the participants were not aware of their BPA exposure levels or hormone levels; hence, the observed association was not likely to be explained by selection bias. Fourth, we did not measure the concentrations of other hormones such as estradiol, SHBG, prolactin, or inhibin B, so we could not display the whole picture of effects of BPA on the male hormone profile.

## Conclusion

In conclusion, environmental BPA exposure was associated with an increased serum LH level, especially among male smokers and men with BMI <25 kg/m^2^. BPA levels were also associated with a higher serum FSH level among current smokers and a lower serum total T level among men with BMI ≥25 kg/m^2^. These findings suggest that the effects of environmental BPA exposure on hormone levels in men might be modified by smoking and BMI.

## Supporting Information

S1 Table Associations between urinary bisphenol A (BPA) and serum hormones among men without history of exposure to chemicals. (DOCX)

## Abbreviations

BMI: Body mass index
BPA: Bisphenol A
CI: Confidence interval
CV: Coefficient of variation
FSH: Follicle-stimulating hormone
GM: Geometric means
HPLC: High-performance liquid chromatography
LH: Luteinizing hormone
LOD: The limit of detection
RR: Relative risk
SHBG: Sex Hormone Binding Globulin
T: Testosterone
