# Supplementary material for: The Association between Exposure to Environmental Bisphenol A and Gonadotropic Hormone Levels among Men
Source: PLoS One. 2017 Jan 13;12(1):e0169217. doi: 10.1371/journal.pone.0169217 (PMC5234835; doi:10.1371/journal.pone.0169217)
Supplement: S1 Table — (DOCX) [file pone.0169217.s001.docx]

**S1 Table. Associations between urinary bisphenol A (BPA) and serum hormones among men without history of exposure to chemicals^a^**

|  | **BPA detection** | N | %  (Hormone>P_75_) | Crude RR  (95%CI) | Adjusted RR ^b^  (95%CI) |
| --- | --- | --- | --- | --- | --- |
| FSH(IU/L) |  |  |  |  |  |
|  | No | 152 | 22.4 | Ref | Ref |
|  | Yes | 364 | 26.9 | 1.20(0.86-1.69) | 1.25(0.89-1.75) |
|  | Lowest tertile^C^ | 121 | 25.6 | 1.12(0.74-1.70) | 1.21(0.80-1.83) |
|  | Middle tertile | 117 | 24.8 | 1.09(0.71-1.66) | 1.19(0.78-1.82) |
|  | Highest tertile | 116 | 30.2 | 1.32(0.89-1.96) | 1.21(0.82-1.81) |
| LH(IU/L) |  |  |  |  |  |
|  | No | 152 | 17.1 | Ref | Ref |
|  | Yes | 364 | 25.3 | **1.48(0.99-2.19)** | **1.48(1.00-2.19)** |
|  | Lowest tertile | 121 | 24.0 | 1.39(0.87-2.20) | 1.45(0.91-2.30) |
|  | Middle tertile | 117 | 24.8 | 1.43(0.90-2.28) | 1.39(0.87-2.22) |
|  | Highest tertile | 116 | 27.6 | 1.60(1.02-2.50) | 1.48(0.94-2.34) |
| T(ng/mL) |  |  |  |  |  |
|  | No | 152 | 29.6 | Ref | Ref |
|  | Yes | 364 | 25.0 | 0.84(0.62-1.14) | 0.80(0.60-1.08) |
|  | Lowest tertile | 121 | 30.6 | 1.05(0.73-1.51) | 0.98(0.68-1.40) |
|  | Middle tertile | 117 | 23.1 | 0.80(0..53-1.20) | 0.87(0.59-1.29) |
|  | Highest tertile | 116 | 21.6 | 0.74(0.49-1.13) | 0.69(0.45-1.04) |

^a^ FSH: Follicle-stimulating hormone, LH: Luteinizing hormone, T: Total testosterone

^b^ RR: relative risk; adjusted for age, BMI, nationality, alcohol intake, and history of chemical exposure.

^C^ The sum of 3 tertiles was 10 less than the number of BPA exposed men due to 10 missing value in creatinine-adjusted BPA concentration.
